# Studying feasibility and effects of a two-stage nursing staff training in residential geriatric care using a 30 month mixed-methods design [ISRCTN24344776]

**DOI:** 10.1186/1472-6955-10-10

**Published:** 2011-05-31

**Authors:** Elsbeth Betschon, Michael Brach, Virpi Hantikainen

**Affiliations:** 1 Institute of Applied Nursing Sciences IPW-FHS, St. Gallen University of Applied Sciences, FHS St. Gallen, Rosenbergstrasse 22, Postfach 627, CH-9001 St. Gallen, Switzerland; 2 Institute of Sport and Exercise Science, University of Muenster, Horstmarer Landweg 62b, 48149 Muenster, Germany

## Abstract

**Background:**

Transfer techniques and lifting weights often cause back pain and disorders for nurses in geriatric care. The Kinaesthetics care conception claims to be an alternative, yielding benefits for nurses as well as for clients.

Starting a multi-step research program on the effects of Kinaesthetics, we assess the feasibility of a two-stage nursing staff training and a pre-post research design. Using quantitative and qualitative success criteria, we address mobilisation from the bed to a chair and backwards, walking with aid and positioning in bed on the staff level as well as on the resident level. In addition, effect estimates should help to decide on and to prepare a controlled trial.

**Methods/Design:**

Standard basic and advanced Kinaesthetics courses (each comprising four subsequent days and an additional counselling day during the following four months) are offered to n = 36 out of 60 nurses in a residential geriatric care home, who are in charge of 76 residents. N = 22 residents needing movement support are participating to this study.

On the staff level, measurements include focus group discussions, questionnaires, physical strain self-assessment (Borg scale), video recordings and external observation of patient assistance skills using a specialised instrument (SOPMAS). Questionnaires used on the resident level include safety, comfort, pain, and level of own participation during mobilisation. A functional mobility profile is assessed using a specialised test procedure (MOTPA).

Measurements will take place at baseline (T0), after basic training (T1), and after the advanced course (T2). Follow-up focus groups will be offered at T1 and 10 months later (T3).

**Discussion:**

Ten criteria for feasibility success are established before the trial, assigned to resources (missing data), processes (drop-out of nurses and residents) and science (minimum effects) criteria. This will help to make rational decision on entering the next stage of the research program.

**Trial Registration:**

Current Controlled Trials ISRCTN24344776.

## Background

The daily duties of the geriatric nurses include many tasks including patient transfer. They also spend 36% of their working time in awkward postures [1]. This leads to physical strain, especially in patient transfer situations and nurses exhibit a high risk of developing musculoskeletal disorders, particularly in the back. In Germany 25.5% of the absenteeism of nurses in inpatient care is due to musculoskeletal disorders [2].

With increasing age immobility or even a bedridden state can ensue [3]. Due to the limits or lack of personal independence, the subjective well-being is restricted [4]. If a person is moved without being able to participate in the action, a feeling of helplessness is likely to arise or the person is pushed into an even worse level of disability. This means that the functional disability of the patient appears greater than it really is because of the deterioration in their physical or psychological condition [5,6]. This induces a feeling of helplessness and the patient does not know, as a result, which abilities they still really have command over [7]. The development and improvement of movement competence can influence positively the patient's well-being and health [8]. Fortunately, movement competence can also be improved even in old age [9].

Kinaesthetics, a care conception developed by Hatch and Maietta [10], aims to increase nurses' movement support skills relating to residents' daily activities and offers advantages both for nurses and residents. In order to implement this conception into practice, a training-on-the-job approach is favoured.

In the present paper, we

• introduce Kinaesthetics and show the lack of research evidence for the benefits of Kinaesthetics in geriatric care

• conclude that there is need for a strict and multi-step research programme, that considers complexity of this kind of intervention,

• describe the study protocol of the first trial in our programme, a pilot in order to check feasibility of a training programme for nurses as well as feasibility of learned skills implementation for nurses and for residents in clinical practice.

### Introduction to Kinaesthetics and review of effectiveness studies

#### The Kinaesthetics conceptual framework

Kinaesthetics is a care concept for human movement, which describes and analyses the fundamental nature of human movement with regard to self-control, functioning and health development. The theoretical bases are to be found in behavioural cybernetics, human psychology and various directions and styles of modern dance [10]. The founders of Kinaesthetics are Dr. L. Maietta and Dr. F. Hatch, USA, who first used Kinaesthetics with students who were interested in their own movement skills. The core of Kinaesthetics is the communication and interaction between humans which is carried out while moving [10]. This human interaction is considered under six concepts; interaction, functional anatomy, human movement, human functions, effort expended as a means of communication, and the environment. The sub-themes of the concepts allow a deeper analysis into different aspects of movement. Each of these concepts is present in every interaction and they can be used for a systematic analysis of human movement resources (Table 1) [10].

**Table 1:** The concepts of Kinaesthetics

Main concepts	Sub-themes
1 Interaction	Senses (visual, auditory, taste, smell, tactile, kinaesthetic)
	Movement elements (time, effort, space)
	Types of interaction (mutual, serial, unilateral)
2 Functional anatomy	Bones and muscles
	Masses and spaces
	Orientation to one's own body
3 Human movement	Postural and transport movement
	Parallel and spiral movement patterns
4 Effort	Pulling and pushing
5 Human functions	Simple human functions (positions)
	Complex human functions (locomotion and movement in place)
6 Environment	Movement supporting and restricting environment

#### Conception for nurse education in Kinaesthetics

The purpose of Kinaesthetics training is to give nurses the fundamental understanding of natural human movement and of its meaning with regard to human existence, perception of one's own body, environment and functioning in daily life. Through this understanding the nurses should develop their own movement competences.

Movement competence is defined as the ability to use one's own movement for solving motor, cognitive or social challenges with motion and to create optimal situations [9]. Ideally, it uses all of the internal and external resources for motor situations in daily life such as in their job and leisure time. Internal resources are the motor abilities and skills, e.g. physical condition, coordination, cognition and constitution. External resources are the potential of the environment such as devices or the social environment [11]. Nurses are more able to work in a resource-orientated manner, to perform different resident movement tasks with ease, to support the patient's active participation towards their own locomotion and to increase the patient's perceptions of their own body and environment. The methods of teaching are interpersonal and interactive.

In Kinaesthetics courses, nurses learn first to understand each concept in their own body. They learn to recognise what happens in their own body while moving themselves. This is a precondition for their understanding of the movement of other people. Secondly, they learn to move other people by using their experiences of each concept. Thirdly, they use Kinaesthetics concepts in different patient situations. The workbook is used to support the cognitive learning.

#### Studies on the effects of Kinaesthetics for the nursing staff

There are only few studies of the effects of Kinaesthetics on the nursing staff. One study in Finland investigated the impact of Kinaesthetics on the physical strain of the nurses in one health care centre taking care of elderly surgical and medical patients [12]. The aim of this study was to show the change in the physical strain on back and shoulder muscles in 12 nurses who swapped the usual transfer technique for the new method, Durewall^® ^(a transfer technique from Sweden) and Kinaesthetics 83 transfers (bed-wheelchair/wheelchair-bed) were videotaped (25 before training, 28 after the first training and 30 after the second training). During the transfers, the nurses' activity of m. trapezius and m. erector spinae were measured on both sides with a portable EMG.

Furthermore, experts in Kinaesthetics evaluated the nurses' individual performance and learning in patient transfer tasks and the patient participation in locomotion activities from the videotapes. For this purpose a newly developed instrument, SOPMAS (Structure of The Observed Patient Movement Assistance Skill) was used. It covers the following ergonomic and Kinaesthetics items: interaction, patient's movement, nurse's posture and movements as well as environment and auxiliary devices. The rating scale ranged from 1 = no skills to 5 = very good skills.

The results indicated, that the nurses could develop their own movement competence and that the mobilisation with Durewall^® ^or Kinaesthetics reduced the physical load by 50%. The EMG results correlated significantly with the SOPMAS results, which means, that a higher SOPMAS score signifies a decline in physical strain [12].

In a prospective, descriptive comparative study, Burns and Sailer investigated whether Kinaesthetics can positively influence the discomfort in neck and lumbar spine by nurses in a neurological clinic [13]. In the intervention group there were 65 nurses from 6 wards. In the control group there were 60 nurses from 6 wards. The answers from the questionnaire showed that the intervention group which was trained in Kinaesthetics had less back pain than the control group. These results indicate that training in Kinaesthetics could reduce the back pain in nursing staff.

#### Studies on the effects of Kinaesthetics for the elderly residents

In the study of Tamminen-Peter (2005) patients were asked for their safety, control and comfort during the mobilisation with a bipolar scale from -4 to +4. All of these three quality indicators were found to be significantly better by the mobilisation with the new methods (Durewall and Kinaesthetics) [12].

One case study examined if a movement support based on Kinaesthetics furthers the development and improvement of body awareness, movement abilities and functional independence of elderly nursing home residents [14]. Two elderly nursing home residents, who showed a strong dependence (Barthel index II; 20-60 points) after a stroke, demonstrated a clear improvement in information understanding, body perception, and movement abilities. Functional independence measured by Barthel index increased in case A from 30 to 40 points and, case B from 55 to 95 points. The results indicate that movement support based on the principles of Kinaesthetics, if used consistently, continuously and in a manner adapted to the situation of the person requiring assistance, could increase the body perception, movement abilities, and the functional independence of patients of advanced age.

Burns and Sailer studied patient's independence in the activity of daily-life movement and how the patients experienced the concept of Kinaesthetics [13]. Twelve patients were interviewed in a semi-structured interview. The result was that the patient experienced a higher feeling of safety and independence. It is not clear, however, if it was the concept of Kinaesthetics or the greater attention of the nurses which influenced the results with regard to increased feeling of safety and greater independence on the part of the patients. One further limitation was the fact that the nurses were not all equally adept in the use of the concept of Kinaesthetics.

On the whole, health care organisations are investing heavily in staff training in Kinaesthetics and the experience from clinical practice is very positive. Nevertheless, we have a lack of research evidence of the effectiveness of Kinaesthetics training for nursing staff and patients.

### Program theory and research plan

In order to make comprehensive evaluations possible, explicating the theory of a programme is helpful and often recommended. This is particularly true for complex interventions including exercise and motor learning [15,16]. Logical models can be used to form a programme theory.

**Figure 1:**
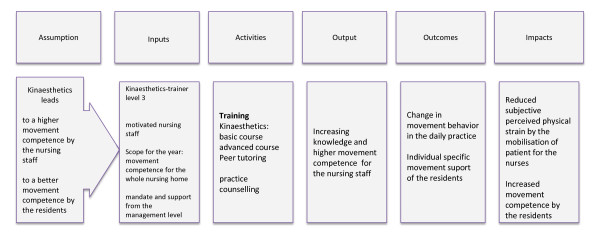
**Logical model of the Kinaesthetics training intervention for trial**.

According to Kellogg Foundation [17] we developed a logical model (Figure 1) connecting the following key elements of the intervention: assumption, inputs, activities, output, outcomes and impacts.

The assumption is that Kinaesthetics training leads to a better movement competence of the nursing staff. A change in movement behaviour takes place with an individual specific movement support of the residents. This leads to a reduction in the subjectively perceived strain of the nurses and to a higher movement competence of the residents.

Using this model, a feasibility study is conducted. The study protocol will be prescribed in the subsequent sections.

### Reasons and objectives of the pilot trial and criteria of feasibility

Conceptualising the feasibility trial, we followed the tutorial Thabane and co-workers propounded [18]. The pilot is to cover several objectives on the staff level and on the resident level. The objectives are assigned to three main reasons for conducting a pilot: there are resources-oriented, scientific-oriented and process-oriented reasons. As used by another work group [19,20], main reasons, feasibility objectives and questions as well as criteria for feasibility success are summarised in Table 2. There are three resources-oriented criteria, five scientific-oriented criteria and two process-oriented criteria of feasibility success.

**Table 2:** Resources, scientific and process criteria

Main reason for conducting a pilot study	Feasibility objective	Feasibility questions	Programme level	Criterion for feasibility success
Resources	Efforts for and completeness of recording data	Are the efforts for the participation in the trial reasonable? Is enough data obtained for a reasonable decision-making? Are the expenses justifiable?	residents	Less than 50% the residents would not participate again in the trial or drop out during the trial (RES_1)
			nurses	Less than 50% the nurses would not participate again in the trial or drop out during the trial (RES_2)
			both	Percentage of missing values below 20% for SOPMAS, below 50% for MOTPA (RES_3)
Scientific	Minimum effects (justifying further research)	Is Kinaesthetics mobilisation able to increase safety, participation and comfort as well as decrease pain for the residents?	residents	After staff training, at least 50% of the residents (a) perceive more safety, comfort and participation during mobilisation (at least 2 points, SCI_1) (b) perceive less pain during mobilisation (at least 2 points) SCI_2 (c) receive a higher median SOPMAS score of item "patient movement" (at least 1 point) (SCI_3)
		Is Kinaesthetics mobilisation able to decrease perceived physical strain and increase movement competence of the nurses ?	nurses	After staff training, at least 50% of the nurses (a) perceive less physical strain during mobilisation (at least 2 points) or increased Borg values can be ascribed to improvements in body perception (SCI_4) (b) receive a higher median SOPMAS score (at least 1 point) (SCI_5)
Process	Continuing Participation	Will residents and nurses be ready to participate in the trial over the full period?	residents	50% or less of the residents leave the programme on their own decision (i.e. not due to an adverse event or death (PRO_1)
			nurses	30% or less of the nurses leave the programme on their own decision (i.e. not due to an adverse event, PRO_2)

## Methods/Design

### Design, ethics approval and registration

This pilot trial is an explorative mixed-methods intervention study with pre-test and post-test. The ethical review board of the cantons Basel Stadt and Basel Land (Switzerland) approved the proposal on the September 16th, 2010 (reference no. 224/10). The trial has been registered at Current Controlled Trials as ISRCTN24344776.

### Setting and Sample

Reasoning on sample and sample size for this pilot, we followed Thabane et al. [18], who recommend orientation at the feasibility criteria (see below). From this point of view, the present study does not allow sample size calculation. Consequently, inference statistics is not intended.

In order to avoid side effects, all eligible residents and all nurses in charge of them should be asked for participation in the study and offered Kinaesthetics training, respectively. On one hand, we considered a nursing home of typical size to yield a sufficient number of participants on the resident as well as the staff level. On the other hand, conducting the trial with two or more centres could be a waste of resources in case of feasibility problems.

The trial is conducted in a nursing home of the canton Basel, Switzerland, with 76 residents and 60 nursing staff. All seniors, who fulfil the inclusion criteria, will be asked to participate. According to the head nurse, 22 residents are eligible at the moment. For this feasibility study, 36 nurses who are regularly in charge are included. Based on the fact that usually about 5% of the persons asked do not wish to participate and 10% will drop out, the final sample size is estimated 19 residents.

Concerning the sample size of the nurses, it is assumed that about 5% of the persons asked that had the Kinaesthetics training would not like to participate and 10% will drop out. Consequently, the final sample size of the nurses is estimated about 31.

On the staff level, inclusion criteria are:

• participation in the Kinaesthetics training course during trial

• no Kinaesthetics training before

• informed consent

On the resident level, inclusion criteria are:

• needs assistance in mobility

• physical condition allows for participation

• can read and speak German

• is able to understand the study information

• informed consent

### Intervention and measurement points

**Figure 2:**
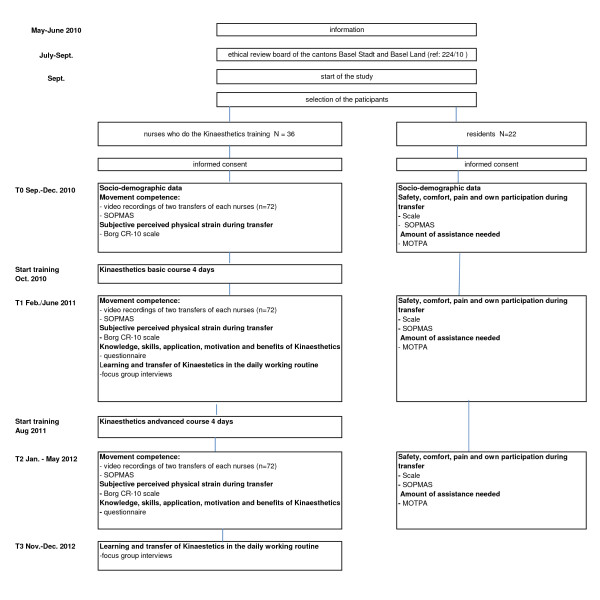
**Flowchart of the study**.

The duration of the study will take 30 months from September 2010 to December 2012, the intervention will start in October 2010. The process and time schedule is shown in Figure 2. Measurements will be taken on the staff level and on the resident level as well. The whole training course and practice counselling will be carried out by an external licensed Kinaesthetics trainer who is not involved in this study.

After the T0 measurement point (pre-test) all nurses will be trained in a basic course in Kinaesthetics for 4 days and have practice counselling of 1 day within 4 months. 36 nurses will be trained in two groups, each of 18 nurses. After this, first post-test (T1) will take place. An advanced course in Kinaesthetics will be held 10 months after the basic course for 4 days within 4 months, before T2. At T0, T1 and T2, mobilisation of patients will be recorded on video, e.g. mobilisation out of bed and from bed to chair and backwards, walking with aid and positioning in bed. At T1 and T3, nurses will be invited to focus group discussions.

### Primary outcome measures

The outcome measures refer to the feasibility criteria described in chapter "Reasons and objectives of the pilot trial and criteria of feasibility". There are measurements on the staff level and on the resident level.

#### Resource-oriented feasibility

At T2, residents as well as staff will be asked if they once more would take part in the study. Using the percentage of negative replies and the drop-out rate, feasibility criteria RES_1 and RES_2 can be calculated. RES_3 (missing values) will be calculated after data processing.

#### Science-oriented feasibility

Immediately after each transfer recorded at T0, T1 and T2, the residents will be asked about the safety, comfort, pain and one's own participation during the transfer with a questionnaire. The scale ranges for pain from 0 = no pain to 5 = unbearable pain; for safety from 0 = very unsafe to 5 = very safe; for comfort from 0 = very uncomfortable to 5 = very comfortable and for participation from 1 = very low participation to 5 = very high participation. The feasibility criteria SCI_1 and SCI_2 will be calculated after data processing.

Immediately after every transfer recorded at T0, T1 and T2 the nurses will be asked about the subjectively perceived physical strain during patient transfer. The data will be collected with the Borg CR10 scale of perceived strain, with a scale from 0 = no strain at all to 10 = extreme strain after every patient transfer (SCI_4) [21].

The movement competence of the nurses will be measured with the SOPMAS Instrument (Structure of the Observed Patient Movement Assistance Skill) (SCI_5). It observes each nurse's individual performance and learning in patient transfer tasks and patient participation in locomotion activities with 4 items: interaction, patient's movement, the nurse's posture and movement, environment and auxiliary devices. The scale ranges from 1 = no skills to 5 = very good skills. All scale levels were given specific definitions, suited for patient transfer situations. The instrument was tested and compared with the Swedish DINO instrument [22] of which validity and reliability have been tested. The correlation was tested by the Pearson test and was good (r = 0.72), though there was a level difference. The highest level of the SOPMAS was missing in the DINO [12]. Video recordings of two transfers of each nurse, e.g. bed-wheelchair/chair-bed, transfer in bed (positioning) will be recorded at the measurement points at the baseline before training (T0), within one month after the basic course (T1) and within one month after the advanced course (T2). The feasibility criteria SCI_3 of the residents for the Item patient's movement and the score over all items SCI_5 for the nurses will be calculated after data processing.

#### Process-oriented feasibility

The reasons of residents and staff leaving the programme will be discriminated. Adverse events (AE) are considered in the process-oriented feasibility. Therefore, the whole number of drop-outs will be diminished by the drop-outs due to AE. Leaving the programme on one's own decision should be 50% or less for the residents (PRO_1), and 30% or less for the nurses (PRO_2).

### Secondary outcome measures

Further outcomes should yield additional information, especially for improvements of the intervention and the research design, and for obtaining information of possible effects. In order to reach this goal, there are quantitative and qualitative measurements on the staff level and on the resident level.

#### Nursing staff level

##### Socio-demographic data

Age, sex, education, profession and musculoskeletal disorders will be recorded with a questionnaire at T0.

##### Knowledge, skills, application, motivation and benefits of Kinaesthetics

A structured questionnaire about knowledge and skills (7 Items, with scaling from totally agree to totally disagree), application (9 items, with scaling from totally agree to totally disagree), motivation (11 items, with scaling from totally agree to totally disagree) and benefits (7 items, with scaling from no benefit to very great benefit) was developed by the primary investigator (V. Hantikainen), Kinaesthetics trainers and peer tutors from Germany, Finland and Switzerland. The content validity of the German version was tested by two Kinaesthetics trainers and seven nurses who were in training as peer tutors. The measurement points are within one month after the basic course (T1) and within one month after advanced course (T2).

##### Learning and transfer of Kinaesthetics in the daily working routine

There will be 2 focus group interviews for the nurses participating in this study with questions about the importance of movement in nursing care, experience with the learning process and the transfer of Kinaesthetics in clinical practice. The measurement point will be after the basic course (T1) and within six months after the advanced course (T3).

#### Resident level

##### Socio-demographic data

Age, sex, movement problems, need for movement assistance, pain medication, participation in the in-house movement group will be recorded with a questionnaire at T0.

##### Functional mobility

The MOTPA instrument (mobility test for residents in acute care) [23,24] observes the amount of assistance needed in 11 functional tasks, which includes

- Lying in the bed: moving to the top, moving sideways, transfer from supine to lateral position, transfer from lateral lying position to sitting on the edge of the bed

- Sitting on the edge of the bed: moving forward, keeping a sitting position, standing up

- Standing position: turning 180°, going backwards 3 steps, short walk (6 meters), walk (30 meters), sitting down.

The mobility profile will be assessed from the same video recordings mentioned above. Measurement points are within one month after basic course (T1) and within one month after advanced course (T2).

## Discussion

This project focuses on the effects of Kinaesthetics training for nurses and residents in nursing homes for elderly people. As part of their training, nurses learn new content working on their own or through group and pair work. The focus of this work centres on nurses own movement competences -- working on the assumption that as practical-based learning is the best way to learn.

Nurses learn as a process, which is characterised by a relatively stable change in their behaviour, thought or sensation based on experiences or newly acquired knowledge [25]. This begs the question whether the requisite learning process is actually taking place and being successfully transferred into practice.

The difficulty of being able to prove the benefits of Kinaesthetics is that the cause interdependencies of this intervention are not linear, but multi-dimensional. On the one hand, the effectiveness of such complex interventions (interventions that have multiple interacting components) cannot be viewed from the sum of its individual parts, thereby making it difficult to evaluate by examining individual factors [26]. On the other hand, there is also the difficulty of transferring the information acquired during training into the next step. Implementing Kinaesthetics in practice requires carers to incorporate both the knowledge they have acquired from their training, as well as any new associated knowledge, into individual care scenarios and their daily work at nursing care [27]. The learning and development process is what is important here, not just learning a care technique. This brings about a change in carers' behaviour in their everyday work. The change in behaviour is a laborious process, which often fails due to a lack of time or the initial amount of extra work required.

## Competing interests

The authors declare that they have no competing interests.

## Authors' contributions

Regarding authorship contributions, EB contributed to the conceptualisation and design of the study, is responsible for data analysis and drafting the article. VH, principal investigator, was mainly responsible for conceptualising and designing the study, for supervising data collection and drafting the article. MB developed the MOTPA mobility test, contributed to the feasibility criteria and was responsible for critically revising the article. All authors read and approved the final manuscript.

## Pre-publication history

The pre-publication history for this paper can be accessed here:

http://www.biomedcentral.com/1472-6955/10/10/prepub
